# Observational study to predict the efficacy and optimal duration of nivolumab treatment in patients with previously treated advanced or recurrent non-small cell lung cancer

**DOI:** 10.1093/jjco/hyac159

**Published:** 2022-10-26

**Authors:** Yasushi Goto, Kiyotaka Yoh, Terufumi Kato, Yukio Hosomi, Kazuhiro Usui, Tomoya Fukui, Katsuya Hirano, Hiroshi Tanaka, Masataka Taguri, Hideo Kunitoh

**Affiliations:** Department of Thoracic Oncology, National Cancer Center Hospital, Chuo-ku, Tokyo, Japan; Department of Thoracic Oncology, National Cancer Center Hospital East, Kashiwa, Chiba, Japan; Department of Thoracic Oncology, Kanagawa Cancer Center, Yokohama, Kanagawa, Japan; Department of Thoracic Oncology & Respiratory Medicine, Tokyo Metropolitan Cancer and Infectious Diseases Center Komagome Hospital, Bunkyo-ku, Tokyo, Japan; Division of Respirology, NTT Medical Center Tokyo, Shinagawa-ku, Tokyo, Japan; Department of Respiratory Medicine, Kitasato University School of Medicine, Sagamihara, Kanagawa, Japan; Department of Respiratory Medicine, Hyogo Prefectural Amagasaki General Medical Center, Amagasaki, Hyogo, Japan; Department of Internal Medicine, Niigata Cancer Center Hospital, Niigata, Niigata, Japan; Department of Data Science, Yokohama City University Graduate School of Data Science, Yokohama, Kanagawa, Japan; Department of Medical Oncology, Japanese Red Cross Medical Center, Shibuya-ku, Tokyo, Japan

**Keywords:** non-small cell lung cancer, nivolumab, quality of life, immune checkpoint inhibitors, patient-reported outcomes

## Abstract

**Background:**

Immune checkpoint inhibitors, including nivolumab, are essential agents for treating non-small cell lung cancer. However, predictive markers are currently lacking, especially using factors based on patient-reported outcomes.

**Methods:**

We conducted a prospective observational study of 244 patients with advanced or recurrent non-small cell lung cancer treated with second- or later-line nivolumab from August 2016 to December 2017. Patient-reported outcomes, including quality of life, were evaluated by the EQ-5D-5L before and during nivolumab treatment. To predict the efficacy of nivolumab during the early treatment phase, we also analyzed the patients’ clinical characteristics, responses and immune-related adverse events at 9 weeks of therapy. The primary endpoint was the disease control rate at 25 weeks after the initiation of nivolumab.

**Results:**

The objective response and disease control rates at 25 weeks were 18.5 and 41.2%, respectively. The emergence of immune-related adverse events at 9 weeks did not significantly affect the disease control rate at 6 months. The response at 9 weeks and patient-reported quality of life were potentially predictive of disease control at week 25. Disease control on week 9 and patients-reported outcomes were potential predictive factors for the overall survival.

**Conclusions:**

This study found no new baseline factors predicting the outcome of nivolumab treatment in patients with non-small cell lung cancer, but response to nivolumab was a robust predictor of overall efficacy. In addition, patient-perceived quality of life could predict the durable efficacy of immune checkpoint inhibitors.

## Teaser

Patient-perceived quality of life could be a predictive factor for the durable efficacy of immune checkpoint inhibitors in patients with non-small cell lung cancer treated with second- or later-line nivolumab.

## Introduction

Immune checkpoint inhibitors (ICIs) have been implemented in the treatment of advanced or recurrent non-small cell lung cancer (NSCLC) since 2015, and they have now become a mainstream treatment option. However, only 10–20% of patients with NSCLC achieve a good response following treatment with single-agent programmed cell death protein 1/programmed death-ligand 1 (PD-L1) inhibitors, while some patients with no apparent cytoreductive efficacy may still show clinical efficacy in terms of survival. Ongoing research has so far failed to identify any definitive factor predicting a response to ICIs. PD-L1 protein expression is currently the best-established biomarker, with ICIs showing clinical efficacy in patients with high PD-L1 expression compared with traditional first-line chemotherapy. Other biomarkers, including tumor mutation burden, tumor microenvironment, immune profiling of tumors or patients and other promising factors, have failed to become clinically established.

Given that ICIs are now applicable for most patients with NSCLC, it is particularly important to identify those patients most likely to benefit from continuing ICI therapy. Furthermore, the occurrence of pseudo-progression makes clinicians reluctant to discontinue ICIs even in patients with early progression (1,2). Treatment may result in the infiltration and accumulation of immune reactive cells in the tumor, but there is currently no confirmed modality for distinguishing this from tumor progression. The high expectations of ICIs and their relative lack of side effects mean that patients may continue to receive unnecessary doses of ICI.

There is thus a need to identify predictive markers suitable for clinical use. Immune-related adverse events (irAEs) are regarded as predictive of an ICI response (3–5). Concerns that longer treatment is more likely to cause subsequent adverse events can be challenged by landmark analysis or by showing that the adverse event precedes the tumor response. Quality of life (QoL) and other patient-reported outcomes (PROs) should also be evaluated, given that they are known predictive factors in relation to traditional chemotherapies (6,7).

**Figure 1:**
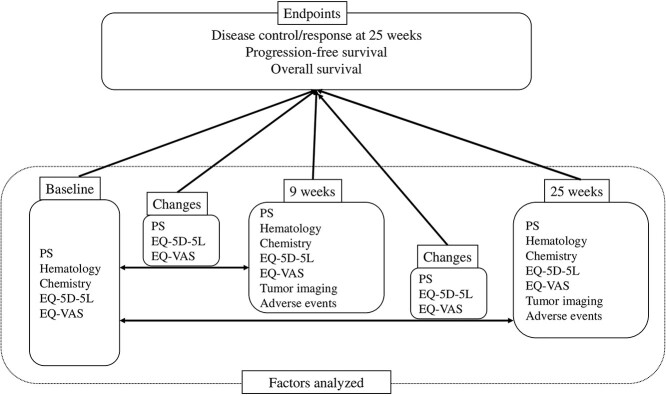
Scheme of the study. Clinical characteristics and patient-reported outcomes at baseline, 9 weeks and 25 weeks, and differences in factors evaluated for endpoints. PS, performance status; VAS, visual analog scale.

In this study, we aimed to investigate the predictive value of responses in patients with advanced NSCLC treated with single-agent nivolumab for second- or later-line chemotherapy. To better understand the predictive course, we used a dynamic prediction model incorporating both baseline characteristics and clinical factors at various time points to evaluate the prediction of longer-term outcomes (Fig. 1). Clinical response, irAEs and PROs measured by the EQ-5D health questionnaire were evaluated.

## Patients and methods

### Study design

This was a prospective observational study of patients with NSCLC who received nivolumab as second- or later-line therapy. Nivolumab was provided in accordance with daily practice. Nivolumab was initially administered at 2 mg/kg every 2 weeks and subsequently at 240 mg/body every 2 weeks. Patients were encouraged to undergo radiological evaluations every 8 weeks until 24 weeks. PROs were evaluated using the EQ-5D-5L system for QoL before nivolumab initiation and at the 3rd (5 weeks), 5th (9 weeks), 7th (13 weeks) and 13th (17 weeks) of administration (Supplementary Table 1).

### Patient selection

Patients aged ≥20 years with histologically or cytologically diagnosed NSCLC were recruited. At the time of study, nivolumab was indicated for patients who had received at least one cytotoxic chemotherapy regimen, but the number of previous regimens was not limited. Adjuvant chemotherapy or chemoradiotherapy regimens completed >2 years before the current nivolumab treatment were not included in the number of previous regimens. Patients with active epidermal growth factor receptor gene mutation and anaplastic lymphoma kinase translocation were excluded. Patients who were unable to answer the QoL survey and those receiving >10 mg prednisolone or an equivalent steroid dose with another formula were also excluded. All patients provided written consent. This study was approved by the institutional review board of each participating institution and by the ethics committee of the Public Health Research Foundation (UMIN000023131).

### Survey and clinical outcomes

Both radiological and clinical progressions (if nivolumab was administered beyond progression) were reported by each physician. Clinical progression was categorized as one or more of the following: (i) emergence or worsening of clinical symptoms due to disease progression; (ii) deterioration of Eastern Cooperative Oncology Group performance status (PS) due to disease progression; (iii) any threat to major organs (such as lymphangitis carcinomatosa, spinal cord compression, carcinomatous meningitis or hepatic failure due to liver metastasis) and (iv) unequivocal multi-organ progression with or without symptoms (8).

Clinical biomarkers that could potentially predict the efficacy of nivolumab were analyzed using clinical parameters at week 9 (after four cycles), including laboratory data, PS, irAEs and QoL score.

### Statistical analysis

The primary endpoint was the disease control rate (complete response, partial response and stable disease) at 25 weeks after the initiation of nivolumab treatment. Secondary endpoints included response rate (complete response and partial response without confirmation), overall survival (OS), 1- and 2-year survival rates, progression-free survival (PFS), clinical PFS (defined as duration from initiation of nivolumab therapy to clinical progressive disease), frequency of irAEs and change of EQ-5D during treatment. Special interest was paid to pulmonary toxicity.

We explored the predictive factors for disease control and response at 25 weeks by univariate and multivariate (for factors with *P* < 0.05 in univariate analysis and possible predictive factors of sex, PS, histology and history of smoking and organ metastasis) logistic regression analyses (Supplementary Table 2). Clinical characteristics before nivolumab initiation and at 9 weeks were analyzed. Predictive factors for OS and PFS were evaluated using a Cox proportional hazards model. Landmark analyses for survival starting from 25 weeks were performed using baseline clinical characteristics and the results of the EQ-5D-5L survey at 25 weeks as covariates. Potential clinical biomarkers included patient characteristics, laboratory data, PS and QoL score before nivolumab initiation and at week 9 (after 4 cycles) and irAEs at week 9.

**Table 1:** Patient characteristics (*n* = 243)

Characteristic	*n* (%) or mean (range)
Sex	
Male	193 (79.4)
Female	50 (20.6)
Age, years	67.7 (32–85)
BMI	18.0 (11–28)
Stage	
Recurrence	60 (24.7)
IV	141 (76.2)
IIIB	23 (12.4)
IIIA	21 (11.4)
Histology	
Adenocarcinoma	148 (60.9)
Squamous cell	80 (32.9)
NOS	10 (4.1)
Other	5 (2.1)
Number of previous regimens	
1	175 (72.3)
≥2	67 (27.7)
PS	
0	72 (29.6)
1	149 (61.3)
2	19 (7.8)
3	3 (1.2)
4	0 (0.0)
Unknown	0 (0.0)
History of surgical resection	
None	203 (83.5)
Yes	40 (16.5)
History of radiotherapy	
None	131 (53.9)
Yes	112 (46.1)
Smoking history	
None	30 (12.3)
Current	9 (3.7)
Ex-smoker	204 (84.0)
Unknown	0 (0.0)
Co-morbidity	
None of the below	156 (64.2)
Liver	5 (2.1)
COPD	74 (30.5)
Interstitial lung disease	8 (3.3)
T factor	
T1a	18 (7.4)
T1b	21 (8.6)
T2a	43 (17.7)
T2b	22 (9.1)
T3	46 (18.9)
T4	62 (25.5)
Unknown	7 (2.9)
Other	24 (9.9)
N factor	
N0	47 (19.3)
N1	25 (10.3)
N2	89 (36.6)
N3	74 (30.5)
Unknown	7 (2.9)
Other	1 (0.4)

*(Continued)*

**Table 1:** Continued

Characteristic	*n* (%) or mean (range)
M factor	
M0	78 (32.1)
M1a	57 (23.5)
M1b	88 (36.2)
Unknown	7 (2.9)
Other	13 (5.3)
Site of metastasis	
Brain	53 (21.8)
Leptomeningeal	0 (0.0)
Bone	52 (21.4)
Liver	19 (7.8)
Adrenal	31 (12.8)
Lung	74 (30.5)
Pleura	46 (18.9)
Pleural effusion	37 (15.2)
Pericardial effusion	7 (2.9)
Other	39 (16.0)
None	38 (15.6)
Proportion of PD-L1 expression in tumors	
<1%	28 (33.7)
≥1%, <50%	41 (49.4)
50%	14 (16.9)

BMI, body mass index; NOS, not otherwise specified; PS, performance status; COPD, chronic obstructive pulmonary disease; PD-L1, programmed death-ligand 1.

## Results

### Patient characteristics

We analyzed a cohort of 244 patients with advanced or recurrent NSCLC who received nivolumab as second- or later-line treatment from August 2016 to December 2017. One patient did not receive nivolumab treatment and the full analysis set thus included 243 patients. By 2 years after the start of treatment in the last-registered case, 14 patients continued and 229 patients had discontinued treatment (disease progression: 176, adverse events: 42 and others: 10). The patient characteristics are given in Table 1. PD-L1 expression (≥1%) was seen in 66% of patients, but PD-L1 was not tested in 66% of patients.

**Figure 2:**
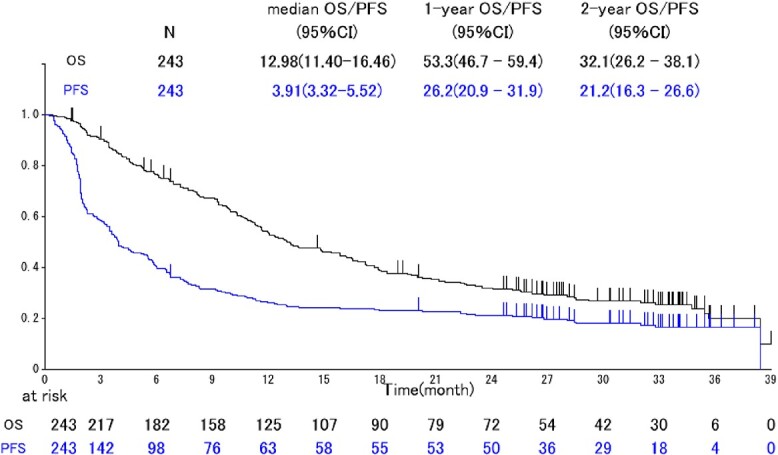
Kaplan-Meier curves of progression-free survival (PFS) and overall survival (OS) in all patients (*n* = 243). CI, confidence interval.

The responses according to the Response Evaluation Criteria in Solid Tumors (RECIST) criteria are shown in Table 2. The median PFS and OS were 3.9 [95% confidence interval (CI): 3.3–5.5] and 12.9 (95% CI: 11.4–16.5) months, respectively (Fig. 2). The compliance of QoL score at 8 weeks was 87% (138/159).

The associations between disease control at week 25 and patient characteristics and clinical data at baseline are shown in Supplementary Table 3. Thirteen patients for whom data were unavailable at week 8 were excluded, and a total of 231 patients were therefore analyzed. N factor (≥N1 vs. N0) and existence of liver metastasis were the baseline factors predicted disease control at week 25.

When patient characteristics and clinical data at week 9, including the occurrence of irAEs, were analyzed, the RECIST-based radiological response at 9 weeks was predictive of disease control at week 25 (Table 3). Exploratory analysis incorporating EQ-5D and EQ-visual analog scale (VAS) scores showed that overall health (EQ-VAS) was also a potential predictive factor of disease control at week 25 (Table 3).

We also analyzed the possible predictive factors for RECIST-based radiological response at 25 weeks, but no baseline characteristics were found to predict the response. However, when clinical characteristics, existence of irAEs, response at 9 weeks and EQ-5D were added for further analysis, the history of smoking, serum albumin, RECIST-based response at 9 weeks and change in anxiety/depression were significant predictive factors of a radiological response at 25 weeks (Supplementary Table 4).

We also evaluated the OS. Among the baseline characteristics, sex, serum ALT, PS (1 vs. 0) at the start of nivolumab treatment and the number of previous treatments (1 vs. ≥2) as well as the liver metastasis and the tumor histology were the significant predictors of better survival (Supplementary Table 5).

We then analyzed QoL and tumor response at 25 weeks, change in QoL and PS within 25 weeks and irAEs up to 9 weeks (*n* = 152) (Supplementary Table 6). Patients without data at 25 weeks (*n* = 91) were excluded. RECIST-based response and irAEs were not predictive of OS, but sex, number of previous treatments, histology, PS (≥2 vs. 0), clinical progression, EQ-5D of usual activities, pain/discomfort and anxiety/depression of week 25 and changes of usual activities were potentially predictive of OS.

The occurrence of irAEs up to 4 weeks (*n* = 243), 8 weeks (*n* = 231) and overall (grades 3 and 4) are listed in Supplementary Tables 7–9. Baseline characteristics and laboratory data were evaluated for the prediction of lung injury, and a higher body mass index and the presence of co-morbidities were identified as the factors potentially predictive of lung injury.

**Table 2:** Response at landmark time

Time	CR *n* (%)	PR *n* (%)	SD *n* (%)	PD *n* (%)	NE *n* (%)	RR *n* [% (95% CI)]	DCR *n* [% (95% CI)]
9 weeks (5th administration)	1(0.4)	42(17.3)	97(39.9)	68(28.0)	35(14.4)	43 [17.7 (13.1–23.1)]	140 [57.6 (51.1–63.9)]
25 weeks (13th administration)	3 (1.2)	42(17.3)	55(22.6)	71(29.2)	72(29.6)	45 [18.5 (13.8–24.0)]	100 [41.2 (34.9–47.6)]
1 year (25th administration)	4 (1.6)	37(15.2)	30(12.3)	40(16.5)	132(54.3)	41 [16.9 (12.4–22.2)]	71 [29.2 (23.6–35.4)]

CR, complete response; PR, partial response; SD, stable disease; PD, progressive disease; NE, not evaluated; RR, response rate; DCR, disease control rate; CI, confidence interval.

## Discussion

This study aimed to identify predictive markers for the durable efficacy of second- or later-line nivolumab. We focused on the clinical conditions, responses and adverse events during treatment and QoL surveys (before and during treatment) to allow the dynamic prediction of the future efficacy of nivolumab treatment in its early phase. The efficacy and toxicity in real-world Japanese NSCLC patients were concordant with previous studies (9,10). As expected, the RECIST-based response at 9 weeks predicted the ongoing response at 25 weeks, and the lack of clinical progression at 25 weeks was predictive of OS, which is in line with a recent meta-analysis showing that response and disease control rates at 6 months predicted 1-year survival (11). Exploratory analyses of EQ-5D and EQ-VAS before and during the treatment showed that changes in anxiety/depression were associated with the response at 25 weeks and that usual activities, pain/discomfort and anxiety/depression were associated with OS. Several studies have shown correlations between irAEs and response to programmed cell death protein 1 pathway inhibitors (3,12,13); however, the current study found that irAEs at 9 weeks did not predict the response at 25 weeks or OS.

An original feature of the current study was the dynamic prediction of nivolumab efficacy. It is important to identify pretreatment predictive factors of efficacy to allow an effective choice of the treatment regimen. ICIs have become established as the standard of care for NSCLC, and most patients expect to receive these at some stage of their treatment. However, it is therefore also important to evaluate their efficacy during treatment, given that futile treatment may result in patients losing their chance to receive other treatments. This is a particular concern, given that there is still no way of distinguishing hyper-progression or disease progression, and a false prediction could represent a great disadvantage for the patient. In dynamic prediction, longitudinal data are collected during treatment and analyzed with covariates to provide more accurate outcome models. Changes in circulating tumor DNA or T lymphocytes are examples of dynamic prediction factors (14–16). The current model used both point values and changes in clinical characteristics (PS and laboratory data) together with response and the emergence of irAEs. In addition to the response, worsening anxiety/depression was also predictive of the response at 25 weeks, while the EQ-5D of usual activities and self-care, pain/discomfort and anxiety/depression were potentially predictive of OS. The importance of patient-oriented outcomes is popular, and this study showed that these may play roles in predicting treatment outcomes (17).

Baseline QoL is an established prognostic factor for survival (18). A better QoL is generally expected to reflect a lower disease burden, fewer co-morbidities, less-severe physical symptoms, higher functioning and expected better survival (19). QoL is also associated with the efficacy of subsequent treatments (20,21). Longitudinal evaluation of QoL and more detailed patient reports can be useful for evaluating toxicity (22,23). We evaluated the EQ-5D-5L and VAS before and during treatment. EQ-VAS shows the patient’s general health and was a potential predictive factor for disease control at 24 weeks. The best EQ-5D questionnaire for reflecting the outcome in patients with cancer remains unclear. Given that changes in anxiety/depression, pain/discomfort and anxiety/depression may affect the prognosis, further evaluation of these questionnaires and clinical outcomes are warranted.

In contrast to most reports, our study did not identify the emergence of irAEs as a predictive factor for further efficacy. The relationship between irAEs and efficacy is complex, given that irAEs continue to emerge as treatment continues, and establishing a landmark point is frequently used to address this problem. Early studies set the point relatively early on, such as at 2 or 6 weeks (3,12); however, our aim was to evaluate a longer-term course, and we therefore used 9 weeks as the landmark, when response evaluation was also available. The emergence of irAEs was not predictive of 24-week disease control or OS in multivariate analysis, including response to treatment. Thus although irAEs have repeatedly been related to efficacy, the current study suggests that they might not predict a better outcome in the long-term.

**Table 3:** Logistic regression analysis of disease control at 25 weeks with clinical data at baseline and 9 weeks after initiation of nivolumab therapy

Factor		Univariate				Multivariate			
		Odds ratio	95% CI		*P* value	Odds ratio	95% CI		*P* value
			Lower	Upper			Lower	Upper	
Sex	Female versus male	0.514	0.252	1.047	0.0667	0.667	0.146	3.056	0.6024
Age	Continuous	0.992	0.965	1.019	0.5380	-	-	-	-
BMI	Continuous	1.102	1.009	1.202	0.0300	1.053	0.909	1.220	0.4893
History of smoking	Yes versus no	1.405	0.618	3.195	0.4168	0.607	0.114	3.241	0.5587
Co-morbidity	Yes versus no	0.794	0.460	1.370	0.4074	-	-	-	-
T factor	≥T2 versus T1	0.756	0.369	1.546	0.4428	-	-	-	-
N factor	≥N1 versus N0	2.282	1.128	4.615	0.0217	2.423	0.921	6.376	0.0729
M factor	≥M1 versus M0	0.484	0.272	0.861	0.0135	0.943	0.383	2.325	0.8991
History of surgical resection	Yes versus no	0.964	0.477	1.949	0.9185	-	-	-	-
History of previous radiotherapy	Yes versus no	1.385	0.820	2.338	0.2231	-	-	-	-
Number of previous treatments	≥2 versus 1	0.728	0.403	1.315	0.2926	-	-	-	-
Brain metastasis	Yes versus no	0.899	0.474	1.706	0.7451	1.045	0.325	3.362	0.9406
Bone metastasis	Yes versus no	0.495	0.253	0.969	0.0402	0.966	0.284	3.285	0.9563
Liver metastasis	Yes versus no	0.065	0.008	0.493	0.0082	0.504	0.031	8.241	0.6311
Histology	Squamous versus other	1.121	0.645	1.949	0.6861	0.718	0.286	1.800	0.4795
WBC/μl^a^	Continuous	1.000	1.000	1.000	0.1247	-	-	-	-
Lymphocytes/μl^a^	Continuous	1.000	1.000	1.000	0.5620	-	-	-	-
Albumin g/dl^a^	Continuous	2.468	1.413	4.310	0.0015	1.162	0.346	3.896	0.8081
ALT U/l^a^	Continuous	1.001	0.981	1.022	0.9070	-	-	-	-
ALP U/l^a^	Continuous	0.999	0.998	1.000	0.1326	-	-	-	-
Cr mg/dl^a^	Continuous	1.028	0.392	2.693	0.9557	-	-	-	-
LDH U/l^a^	Continuous	0.991	0.986	0.996	0.0003	0.994	0.984	1.003	0.2074
CRP mg/dl^a^	Continuous	0.854	0.755	0.967	0.0129	0.995	0.763	1.299	0.9716
FT4 ng/dl^a^	Continuous	1.050	0.740	1.490	0.7863	-	-	-	-
irAE^a^	G1–G2 versus none	1.355	0.766	2.398	0.4926	-	-	-	-
	≥G3 versus none	1.106	0.365	3.352	0.9270	-	-	-	-
PS^a^	1 versus 0	0.506	0.263	0.972	0.4286	0.777	0.275	2.190	0.8661
	≥2 versus 0	0.152	0.053	0.434	0.0016	0.729	0.054	9.825	0.8735
PS change^a^	Continuous	0.531	0.335	0.842	0.0072	1.563	0.569	4.296	0.3863
RECIST	CR, PR versus SD, PD, NE	8.476	3.714	19.345	<.0001	-	-	-	-
	CR, PR, SD versus PD, NE	35.148	13.348	92.548	<.0001	20.187	4.988	81.694	<.0001^b^
Clinical PD^a^	PD, NE versus non-PD	0.055	0.016	0.182	<.0001	0.581	0.068	4.989	0.6208
Mobility^a^	Continuous	0.631	0.458	0.871	0.0051	0.759	0.312	1.845	0.5429
Self-care^a^	Continuous	0.711	0.484	1.043	0.0814	-	-	-	-
Usual activities^a^	Continuous	0.673	0.487	0.929	0.0159	0.943	0.397	2.242	0.8946
Pain/discomfort^a^	Continuous	0.682	0.484	0.961	0.0286	0.706	0.355	1.406	0.3225
Anxiety/depression^a^	Continuous	0.598	0.391	0.914	0.0174	0.591	0.244	1.434	0.2449
Health state^a^	Continuous	1.020	1.005	1.035	0.0098	0.959	0.923	0.996	0.0287^b^
ΔMobility^a^	Continuous	0.898	0.640	1.258	0.5310	-	-	-	-
ΔSelf-care^a^	Continuous	0.896	0.603	1.332	0.5872	-	-	-	-
ΔUsual activities^a^	Continuous	0.914	0.666	1.255	0.5789	-	-	-	-
ΔPain/discomfort^a^	Continuous	0.998	0.724	1.375	0.9885	-	-	-	-
ΔAnxiety/depression^a^	Continuous	0.651	0.435	0.973	0.0363	1.001	0.550	1.824	0.9972
ΔHealth state^a^	Continuous	1.008	0.991	1.025	0.3696	-	-	-	-

WBC, white blood cell count; ALT, alanine aminotransferase; ALP, alkaline phosphatase; Cr, creatinine; LDH, lactate dehydrogenase; CRP, C-reactive protein; FT4, thyroxine; irAE, immune-related adverse events; RECIST, Response Evaluation Criteria in Solid Tumors; CR, complete response; PR, partial response; SD, stable disease; PD, progressive disease; NE, not evaluated, Δ, changes between baseline to week 9.

^a^Evaluated at 9 weeks.

^b^Significantly positive.

This study had several limitations. Nivolumab is currently not a standard second-line treatment for NSCLC since ICIs have been installed for first-line treatment. However, many patients do not have access to frontline ICI treatment for financial reasons. In addition, the results of this study have important economic implications by allowing potentially useless treatment to be stopped after its initiation. Until now, PD-L1 expression in tumor cells has been widely used as a biomarker for ICIs, but more than a quarter of patients in the current study were not tested for the PD-L1 expression. Nivolumab was approved rapidly in Japan, and PD-L1 testing was not required. In addition, PD-L1 expression is useful for predicting efficacy before but not after the start of treatment, and the initial response should be added to the predictive factors in the future. We explored many factors, including clinical characteristics, laboratory data and QoL before and during treatment, as well as incremental changes, in our prediction model. However, multiplicity is unavoidable and cofounding factors are likely to exist. This study was thus a basic exploration of many candidate predictive factors, and further studies based on the results are needed, including incorporating QoL as a potential predictive factor in the updated clinical situation.

In summary, we explored and analyzed the factors predicting a durable response to second- or later-line nivolumab treatment in patients with NSCLC. We incorporated clinical characteristics, response and the emergence of irAEs during treatment in order to better predict disease control and OS. Although no new baseline factors predicting outcome were identified, an initial response to treatment was robustly predictive of the outcome. The emergence of irAEs at 9 weeks did not independently predict the efficacy of nivolumab, but general health QoL and some aspects of the EQ-5D questionnaire may help to evaluate the potential long-term benefit of nivolumab in previously treated patients with NSCLC.

## Supplementary Material

Supplementary_Tables_20220717_hyac159
