# Supplementary material for: Observational study to predict the efficacy and optimal duration of nivolumab treatment in patients with previously treated advanced or recurrent non-small cell lung cancer
Source: Jpn J Clin Oncol. 2022 Oct 26;53(2):153–60. doi: 10.1093/jjco/hyac159 (PMC9885731; doi:10.1093/jjco/hyac159)
Supplement: Supplementary_Tables_20220717_hyac159 [file supplementary_tables_20220717_hyac159.doc]

**Supplementary Table 1**. Schedule of activities

|  | **Baseline** | **Week 5** | **Week 9** | **Week 13** | **Week 17** | **Week 25** | **Week 52** |
| --- | --- | --- | --- | --- | --- | --- | --- |
| Nivolumab administration |  | 3^rd^ | 5^th^ | 7^th^ | 9^th^ | 13^th^ |  |
| Demography* | X |  |  |  |  |  |  |
| Characteristics** | X |  |  |  |  |  |  |
| PS | X | X | X | X | X | X | X |
| Hematology/chemistry | X | X | X | X |  | X |  |
| Tumor imaging | X |  | X |  | X | X | X |
| Adverse events |  | X | X | X | X |  |  |
| EQ-5D-5L, EQ-VAS | X | X | X | X |  |  |  |

*Age, sex, **tumor type, TNM stage, prior anti-cancer therapy, comorbidity, past medical history. PS: performance status; VAS: visual analog scale.

**Supplementary Table 2.** Endpoints and factors analyzed

|  | | **S3** | **T3** | **S4** | **S5** | **S6** |
| --- | --- | --- | --- | --- | --- | --- |
| Disease control | week 25 | EP | EP |  |  |  |
| RECIST response | week 9 |  | X |  |  | X |
|  | week 25 |  |  | EP |  |  |
| Clinical progression (week 9) | |  | X | X |  | X |
| OS | |  |  |  | EP | EP |
| PFS | RESIST PD |  |  |  |  | X |
|  | Clinical PD |  |  |  |  | X |
| Demography | | X | X | X | X | X |
| Characteristics | | X | X | X | X | X |
| Adverse events at 9 weeks | |  | X | X |  | X |
| PS | BL | X |  |  | X |  |
|  | week 9 |  | X | X |  |  |
|  | week 25 |  |  |  |  | X |
| PS change | BL to week 9 |  | X | X |  |  |
|  | BL to week 25 |  |  |  |  | X |
| Hematology/chemistry | BL | X |  |  | X |  |
|  | week 9 |  | X | X |  | X |
| EQ-5D-5L | week 9 |  | X | X |  |  |
|  | ~~BL to~~ week 25 |  |  |  |  | X |
| Change in EQ-5D-5L | BL to week 9 |  | X | X |  |  |
|  | BL to week 25 |  |  |  |  | X |

S: supplemental table, T: table; EP: endpoint; RECIST: Response Evaluation Criteria in Solid Tumors; OS: overall survival; PD: progressive disease; PFS: progression-free survival; BL: baseline; PS: performance status.

**Supplementary Table 3.** Logistic regression analysis for disease control at week 25 with baseline clinical data

| **Factor** | | **Univariate** | | | | **Multivariate** | | | |
| --- | --- | --- | --- | --- | --- | --- | --- | --- | --- |
|  |  | Odds ratio | 95% CI | | *P* value | Odds ratio | 95% CI | | *P* value |
|  |  |  | Lower | Upper |  |  | Lower | Upper |  |
| Sex | Female vs. male | 0.514 | 0.252 | 1.047 | 0.0667 | 0.530 | 0.181 | 1.552 | 0.2470 |
| Age | Continuous | 0.992 | 0.965 | 1.019 | 0.5380 | - | - | - | - |
| BMI | Continuous | 1.102 | 1.009 | 1.202 | 0.0300 | 1.027 | 0.924 | 1.143 | 0.6186 |
| PS | 1 vs. 0 | 0.419 | 0.234 | 0.749 | 0.8230 | 0.460 | 0.241 | 0.878 | 0.9284 |
|  | ≥2 vs. 0 | 0.148 | 0.039 | 0.562 | 0.0239 | 0.228 | 0.052 | 0.993 | 0.1279 |
| History of smoking | Yes vs. no | 1.405 | 0.618 | 3.195 | 0.4168 | 0.930 | 0.263 | 3.293 | 0.9108 |
| Comorbidity | Yes vs. no | 0.794 | 0.460 | 1.370 | 0.4074 | - | - | - | - |
| T factor | ≥T2 vs. T1 | 0.756 | 0.369 | 1.546 | 0.4428 | - | - | - | - |
| N factor | ≥N1 vs. N0 | 2.282 | 1.128 | 4.615 | 0.0217 | 2.281 | 1.058 | 4.919 | 0.0355 |
| M factor | ≥M1 vs. M0 | 0.484 | 0.272 | 0.861 | 0.0135 | 0.697 | 0.357 | 1.360 | 0.2894 |
| Stage | III vs. I-II | 2.833 | 0.765 | 10.490 | 0.0150 | - | - | - | - |
|  | IV vs. I-II | 1.059 | 0.302 | 3.708 | 0.2243 | - | - | - | - |
| History of surgical resection | Yes vs. no | 0.964 | 0.477 | 1.949 | 0.9185 | - | - | - | - |
| History of previous radiotherapy | Yes vs. no | 1.385 | 0.820 | 2.338 | 0.2231 | - | - | - | - |
| Number of previous treatments | ≥2 vs. 1 | 0.728 | 0.403 | 1.315 | 0.2926 | - | - | - | - |
| WBC/μL* | Continuous | 1.000 | 1.000 | 1.000 | 0.0857 | - | - | - | - |
| Lymphocytes/μL* | Continuous | 1.000 | 1.000 | 1.000 | 0.6802 | - | - | - | - |
| Albumin g/dL* | Continuous | 1.625 | 0.988 | 2.675 | 0.0560 | - | - | - | - |
| ALT U/L* | Continuous | 1.005 | 0.986 | 1.025 | 0.6036 | - | - | - | - |
| ALP U/L* | Continuous | 0.998 | 0.996 | 1.000 | 0.0988 | - | - | - | - |
| Cr mg/dL* | Continuous | 0.896 | 0.380 | 2.117 | 0.8030 | - | - | - | - |
| LDH U/L* | Continuous | 0.995 | 0.992 | 0.999 | 0.0056 | 0.998 | 0.995 | 1.002 | 0.3992 |
| CRP mg/dL* | Continuous | 0.946 | 0.878 | 1.021 | 0.1527 | - | - | - | - |
| FT4 ng/dL* | Continuous | 1.014 | 0.593 | 1.734 | 0.9588 | - | - | - | - |
| Brain metastasis | Yes vs. no | 0.899 | 0.474 | 1.706 | 0.7451 | 0.790 | 0.353 | 1.767 | 0.5654 |
| Bone metastasis | Yes vs. no | 0.495 | 0.253 | 0.969 | 0.0402 | 0.903 | 0.390 | 2.087 | 0.8108 |
| Liver metastasis | Yes vs. no | 0.065 | 0.008 | 0.493 | 0.0082 | 0.105 | 0.013 | 0.871 | 0.0368 |
| Other metastasis | Yes vs. no | 0.619 | 0.357 | 1.071 | 0.0861 | - | - | - | - |
| Histology | Squamous vs. other | 1.121 | 0.645 | 1.949 | 0.6861 | 0.860 | 0.434 | 1.702 | 0.6648 |

CI: confidence interval; BMI: body mass index; PS: performance status; WBC: white blood cell count; ALT: alanine aminotransferase; ALP: alkaline phosphatase; Cr: creatinine; LDH: lactate dehydrogenase; CRP: C-reactive protein; FT4: thyroxine. *Evaluated at 9 weeks.

**Supplementary Table 4.** Logistic regression analysis for wee 25 response with clinical data at week 9 after initiation of nivolumab therapy

| **Factor** | | **Univariate** | | | | **Multivariate** | | | |
| --- | --- | --- | --- | --- | --- | --- | --- | --- | --- |
|  |  | Odds ratio | 95% CI | | *P* value | Odds ratio | 95% CI | | *P* value |
|  |  |  | Lower | Upper |  |  | Lower | Upper |  |
| Sex | Female vs. male | 0.261 | 0.077 | 0.885 | 0.0311 | 0.126 | 0.012 | 1.333 | 0.0852 |
| Age | Continuous | 0.986 | 0.954 | 1.019 | 0.3895 | - | - | - | - |
| BMI | Continuous | 1.064 | 0.957 | 1.184 | 0.2498 | - | - | - | - |
| History of smoking | Yes vs. no | 2.174 | 0.626 | 7.548 | 0.2215 | 0.057 | 0.004 | 0.747 | 0.0291 |
| Comorbidity | Yes vs. no | 0.958 | 0.485 | 1.889 | 0.9004 | - | - | - | - |
| T factor | ≥T2 vs. T1 | 0.784 | 0.326 | 1.891 | 0.5886 | - | - | - | - |
| N factor | ≥N1 vs. N0 | 0.822 | 0.371 | 1.824 | 0.6301 | - | - | - | - |
| M factor | ≥M1 vs. M0 | 0.721 | 0.348 | 1.492 | 0.3780 | - | - | - | - |
| History of surgical resection | Yes vs. no | 1.354 | 0.590 | 3.107 | 0.4752 | - | - | - | - |
| History of previous radiotherapy | Yes vs. no | 1.449 | 0.754 | 2.786 | 0.2654 | - | - | - | - |
| Number of previous treatments | ≥2 vs. 1 | 0.693 | 0.321 | 1.499 | 0.3516 | - | - | - | - |
| Brain metastasis | Yes vs. no | 1.693 | 0.807 | 3.548 | 0.1634 | 0.964 | 0.180 | 5.167 | 0.9657 |
| Bone metastasis | Yes vs. no | 0.613 | 0.255 | 1.470 | 0.2725 | 2.037 | 0.352 | 11.788 | 0.4272 |
| Liver metastasis | Yes vs. no | <0.001 | <0.001 | >999.999 | 0.9709 | <0.001 | <0.001 | >999.999 | 0.9710 |
| Histology | Squamous vs. other | 1.025 | 0.513 | 2.045 | 0.9449 | 2.028 | 0.454 | 9.070 | 0.3548 |
| WBC/μL | Continuous | 1.000 | 1.000 | 1.000 | 0.0757 | - | - | - | - |
| Lymphocytes/μL | Continuous | 1.000 | 1.000 | 1.001 | 0.3330 | - | - | - | - |
| Albumin g/dL | Continuous | 4.438 | 2.025 | 9.730 | 0.0002 | 12.694 | 1.549 | 104.029 | 0.0179 |
| ALT U/L | Continuous | 1.013 | 0.991 | 1.036 | 0.2596 | - | - | - | - |
| ALP U/L | Continuous | 0.999 | 0.997 | 1.001 | 0.2000 | - | - | - | - |
| Cr mg/dL | Continuous | 1.506 | 0.528 | 4.292 | 0.4437 | - | - | - | - |
| LDH U/L | Continuous | 0.991 | 0.984 | 0.998 | 0.0077 | 0.987 | 0.972 | 1.002 | 0.0958 |
| CRP mg/dL | Continuous | 0.777 | 0.621 | 0.973 | 0.0276 | 1.165 | 0.694 | 1.956 | 0.5632 |
| FT4 ng/dL | Continuous | 0.984 | 0.637 | 1.521 | 0.9435 | - | - | - | - |
| irAE | G1-G2 vs. none | 1.129 | 0.559 | 2.280 | 0.5448 | - | - | - | - |
|  | ≥G3 vs. none | 0.703 | 0.149 | 3.319 | 0.5983 | - | - | - | - |
| PS | 1 vs. 0 | 0.419 | 0.206 | 0.854 | 0.7234 | 0.350 | 0.076 | 1.604 | 0.3937 |
|  | ≥2 vs. 0 | 0.127 | 0.027 | 0.589 | 0.0310 | 0.669 | 0.014 | 31.884 | 0.9475 |
| Change in PS | Continuous | 0.603 | 0.346 | 1.052 | 0.0748 | - | - | - | - |
| RECIST | CR, PR vs. SD, PD, NE | 60.788 | 23.408 | 157.861 | <.0001 | 367.989 | 43.526 | >999.999 | <.0001 |
|  | CR, PR, SD vs. PD, NE | 19.727 | 4.643 | 83.816 | <.0001 | - | - | - | - |
| Clinical PD | PD, NE vs. non-PD | 0.130 | 0.030 | 0.557 | 0.0060 | 8.823 | 0.747 | 104.227 | 0.0839 |
| Mobility | Continuous | 0.571 | 0.360 | 0.906 | 0.0173 | 2.048 | 0.496 | 8.455 | 0.3215 |
| Self-care | Continuous | 0.507 | 0.243 | 1.057 | 0.0698 | - | - | - | - |
| Usual activities | Continuous | 0.511 | 0.305 | 0.854 | 0.0104 | 0.207 | 0.040 | 1.056 | 0.0582 |
| Pain/discomfort | Continuous | 0.512 | 0.312 | 0.839 | 0.0080 | 0.645 | 0.185 | 2.253 | 0.4922 |
| Anxiety/depression | Continuous | 0.438 | 0.226 | 0.852 | 0.0150 | 1.160 | 0.249 | 5.399 | 0.8500 |
| Health state | Continuous | 1.032 | 1.010 | 1.055 | 0.0043 | 1.008 | 0.955 | 1.064 | 0.7692 |
| ΔMobility | Continuous | 0.786 | 0.518 | 1.193 | 0.2579 | - | - | - | - |
| ΔSelf-care | Continuous | 0.614 | 0.329 | 1.147 | 0.1262 | - | - | - | - |
| ΔUsual activities | Continuous | 0.748 | 0.505 | 1.109 | 0.1484 | - | - | - | - |
| ΔPain/discomfort | Continuous | 0.764 | 0.518 | 1.126 | 0.1738 | - | - | - | - |
| ΔAnxiety/depression | Continuous | 0.513 | 0.314 | 0.838 | 0.0077 | 0.237 | 0.090 | 0.628 | 0.0038 |
| ΔHealth state | Continuous | 1.014 | 0.993 | 1.035 | 0.1985 | - | - | - | - |

CI: confidence interval; BMI: body mass index; WBC: white blood cell count; ALT: alanine aminotransferase; ALP: alkaline phosphatase; Cr: creatinine; LDH: lactate dehydrogenase; CRP: C-reactive protein; FT4: thyroxine; irAE; immune-related adverse events; PS: performance status; RECIST: Response Evaluation Criteria in Solid Tumors; CR: complete response; PR: partial response; SD: stable disease; PD: progressive disease; NE: not evaluated; Δ: changes between baseline to week 9.

**Supplementary Table 5.** Cox regression analysis for overall survival with clinical data at baseline

| Factor | | Univariate | | | | Multivariate | | | | |  |
| --- | --- | --- | --- | --- | --- | --- | --- | --- | --- | --- | --- |
|  |  | Odds ratio | 95% CI | | *P* value | | Odds ratio | 95% CI | | *P* value | |
|  |  |  | Lower | Upper |  | |  | Lower | Upper |  | |
| Sex | Female vs. male | 1.965 | 1.187 | 3.254 | 0.0087 | | 2.064 | 1.048 | 4.065 | 0.0362 | |
| Age | Continuous | 0.991 | 0.968 | 1.015 | 0.4436 | | - | - | - | - | |
| BMI | Continuous | 0.971 | 0.902 | 1.045 | 0.4248 | | - | - | - | - | |
| PS | 1 vs. 0 | 2.079 | 1.364 | 3.170 | 0.0007 | | 1.816 | 1.126 | 2.930 | 0.0145 | |
|  | ≥2 vs. 0 | 4.090 | 2.569 | 6.510 | <.0001 | | 2.312 | 0.709 | 7.540 | 0.1647 | |
| History of smoking | Yes vs. no | 0.927 | 0.496 | 1.732 | 0.8123 | | 1.377 | 0.567 | 3.342 | 0.4798 | |
| Comorbidity | Yes vs. no | 1.076 | 0.727 | 1.594 | 0.7134 | | - | - | - | - | |
| T factor | ≥T2 vs. T1 | 0.881 | 0.551 | 1.408 | 0.5951 | | - | - | - | - | |
| N factor | ≥N1 vs. N0 | 0.730 | 0.468 | 1.139 | 0.1654 | | - | - | - | - | |
| M factor | ≥M1 vs. M0 | 1.281 | 0.845 | 1.943 | 0.2440 | | - | - | - | - | |
| Stage | III vs. I-II | 0.777 | 0.374 | 1.613 | 0.4983 | | - | - | - | - | |
|  | IV vs. I-II | 1.068 | 0.541 | 2.111 | 0.8488 | | - | - | - | - | |
| History of surgical resection | Yes vs. no | 0.845 | 0.522 | 1.368 | 0.4937 | | - | - | - | - | |
| History of previous radiotherapy | Yes vs. no | 0.909 | 0.612 | 1.348 | 0.6342 | | - | - | - | - | |
| Number of previous treatments | ≥2 vs. 1 | 1.605 | 1.051 | 2.452 | 0.0285 | | 2.064 | 1.048 | 4.065 | 0.0362 | |
| WBC/μL* | Continuous | 1.000 | 1.000 | 1.000 | 0.4929 | | - | - | - | - | |
| Lymphocytes/μL* | Continuous | 1.000 | 0.999 | 1.000 | 0.1475 | | - | - | - | - | |
| Albumin g/dL* | Continuous | 0.699 | 0.466 | 1.047 | 0.0824 | | - | - | - | - | |
| ALT U/L* | Continuous | 0.974 | 0.956 | 0.993 | 0.0075 | | 0.974 | 0.952 | 0.996 | 0.0230 | |
| ALP U/L* | Continuous | 1.001 | 0.999 | 1.003 | 0.2011 | | - | - | - | - | |
| Cr mg/dL* | Continuous | 0.158 | 0.050 | 0.498 | 0.0016 | | 0.625 | 0.194 | 2.012 | 0.4310 | |
| LDH U/L* | Continuous | 1.003 | 1.001 | 1.004 | 0.0022 | | 1.001 | 0.999 | 1.003 | 0.1818 | |
| CRP mg/dL* | Continuous | 1.069 | 1.016 | 1.124 | 0.0106 | | 1.012 | 0.942 | 1.088 | 0.7392 | |
| FT4 ng/dL* | Continuous | 1.091 | 0.354 | 3.364 | 0.8789 | | - | - | - | - | |
| Brain metastasis | Yes vs. no | 0.860 | 0.532 | 1.390 | 0.5373 | | 0.878 | 0.502 | 1.535 | 0.6479 | |
| Bone metastasis | Yes vs. no | 1.281 | 0.755 | 2.175 | 0.3585 | | 1.015 | 0.564 | 1.826 | 0.9596 | |
| Liver metastasis | Yes vs. no | 2.892 | 1.663 | 5.028 | 0.0002 | | 3.248 | 1.399 | 7.544 | 0.0061 | |
| Other metastasis | Yes vs. no | 1.422 | 0.950 | 2.128 | 0.0867 | | - | - | - | - | |
| Histology | Squamous vs. other | 1.428 | 0.970 | 2.104 | 0.0713 | | 1.603 | 1.025 | 2.505 | 0.0385 | |

CI: confidence interval; BMI: body mass index; PS: performance status; WBC: white blood cell count; ALT: alanine aminotransferase; ALP: alkaline phosphatase; Cr: creatinine; LDH: lactate dehydrogenase; CRP: C-reactive protein; FT4: thyroxine.

**Supplementary Table 6.** Cox regression analysis for overall survival with clinical data at week 9 after initiation of nivolumab therapy

| **Factor** | | **Univariate** | | | | **Multivariate** | | | |
| --- | --- | --- | --- | --- | --- | --- | --- | --- | --- |
|  |  | Odds ratio | 95% CI | | *P* value | Odds ratio | 95% CI | | *P* value |
|  |  |  | Lower | Upper |  |  | Lower | Upper |  |
| Sex | Female vs. male | 1.965 | 1.187 | 3.254 | 0.0087 | 13.100 | 1.283 | 133.795 | 0.0300 |
| Age | Continuous | 0.991 | 0.968 | 1.015 | 0.4436 | - | - | - | - |
| BMI | Continuous | 0.971 | 0.902 | 1.045 | 0.4248 | - | - | - | - |
| History of smoking | Yes vs. no | 0.927 | 0.496 | 1.732 | 0.8123 | 1.642 | 0.124 | 21.736 | 0.7065 |
| Comorbidity | Yes vs. no | 1.076 | 0.727 | 1.594 | 0.7134 | - | - | - | - |
| T factor | ≥T2 vs. T1 | 0.881 | 0.551 | 1.408 | 0.5951 | - | - | - | - |
| N factor | ≥N1 vs. N0 | 0.730 | 0.468 | 1.139 | 0.1654 | - | - | - | - |
| M factor | ≥M1 vs. M0 | 1.281 | 0.845 | 1.943 | 0.2440 | - | - | - | - |
| History of surgical resection | Yes vs. no | 0.845 | 0.522 | 1.368 | 0.4937 | - | - | - | - |
| History of previous radiotherapy | Yes vs. no | 0.909 | 0.612 | 1.348 | 0.6342 | - | - | - | - |
| Number of previous treatments | ≥2 vs. 1 | 1.605 | 1.051 | 2.452 | 0.0285 | 6.729 | 1.880 | 24.088 | 0.0034 |
| Brain metastasis | Yes vs. no | 0.860 | 0.532 | 1.390 | 0.5373 | 0.446 | 0.055 | 3.627 | 0.4501 |
| Bone metastasis | Yes vs. no | 1.281 | 0.755 | 2.175 | 0.3585 | 2.104 | 0.632 | 7.005 | 0.2256 |
| Liver metastasis | Yes vs. no | 2.892 | 1.663 | 5.028 | 0.0002 | 30.569 | 0.797 | 1172.606 | 0.0661 |
| Histology | Squamous vs. other | 1.428 | 0.970 | 2.104 | 0.0713 | 4.501 | 1.126 | 17.997 | 0.0334 |
| WBC/μL | Continuous | 1.000 | 1.000 | 1.000 | <.0001 | 1.000 | 1.000 | 1.000 | 0.8012 |
| Lymphocytes/μL | Continuous | 1.000 | 0.999 | 1.000 | 0.0768 | - | - | - | - |
| Albumin g/dL | Continuous | 0.155 | 0.087 | 0.279 | <.0001 | 1.288 | 0.289 | 5.740 | 0.7402 |
| ALT U/L | Continuous | 0.941 | 0.906 | 0.977 | 0.0016 | 0.941 | 0.876 | 1.011 | 0.0966 |
| ALP U/L | Continuous | 1.005 | 1.001 | 1.008 | 0.0054 | 1.006 | 1.000 | 1.012 | 0.0531 |
| Cr mg/dL | Continuous | 0.048 | 0.009 | 0.268 | 0.0005 | 3.383 | 0.168 | 68.098 | 0.4263 |
| LDH U/L | Continuous | 1.007 | 1.003 | 1.011 | 0.0002 | 1.010 | 0.998 | 1.022 | 0.0970 |
| CRP mg/dL | Continuous | 1.257 | 1.166 | 1.356 | <.0001 | 0.976 | 0.689 | 1.382 | 0.8903 |
| FT4 ng/dL | Continuous | 0.152 | 0.020 | 1.184 | 0.0721 | - | - | - | - |
| irAE | G1-G2 vs. none | 1.247 | 0.815 | 1.907 | 0.3093 | - | - | - | - |
|  | ≥G3 vs. none | 1.080 | 0.329 | 3.548 | 0.8995 | - | - | - | - |
| PS | 1 vs. 0 | 2.052 | 1.082 | 3.892 | 0.0277 | 1.900 | 0.451 | 8.009 | 0.3819 |
|  | ≥2 vs. 0 | 22.059 | 9.544 | 50.981 | <.0001 | 939.542 | 12.001 | 73556.14 | 0.0021 |
| Change in PS | Continuous | 2.187 | 1.289 | 3.709 | 0.0037 | 0.957 | 0.176 | 5.220 | 0.9597 |
| RECIST | CR, PR vs. SD, PD, NE | 0.241 | 0.141 | 0.413 | <.0001 | - | - | - | - |
|  | CR, PR, SD vs. PD, NE | 0.295 | 0.196 | 0.444 | <.0001 | 0.698 | 0.133 | 3.661 | 0.6709 |
| Clinical PD | PD, NE vs non-PD | 2.436 | 1.474 | 4.024 | 0.0005 | 10.852 | 1.015 | 116.074 | 0.0486 |
| Mobility | Continuous | 1.988 | 1.551 | 2.547 | <.0001 | 2.677 | 0.592 | 12.109 | 0.2010 |
| Self-care | Continuous | 2.742 | 1.919 | 3.919 | <.0001 | 3.489 | 0.336 | 36.249 | 0.2955 |
| Usual activities | Continuous | 1.933 | 1.542 | 2.421 | <.0001 | 0.038 | 0.003 | 0.425 | 0.0080 |
| Pain/discomfort | Continuous | 2.123 | 1.707 | 2.640 | <.0001 | 6.539 | 1.254 | 34.088 | 0.0258 |
| Anxiety/depression | Continuous | 1.884 | 1.412 | 2.513 | <.0001 | 3.751 | 1.437 | 9.793 | 0.0069 |
| Health state | Continuous | 0.977 | 0.964 | 0.989 | 0.0003 | 1.057 | 0.988 | 1.132 | 0.1071 |
| ΔMobility | Continuous | 1.936 | 1.387 | 2.702 | 0.0001 | 1.028 | 0.319 | 3.309 | 0.9631 |
| ΔSelf-care | Continuous | 2.522 | 1.060 | 5.996 | 0.0364 | 0.377 | 0.068 | 2.090 | 0.2641 |
| ΔUsual activities | Continuous | 1.694 | 1.023 | 2.803 | 0.0404 | 6.365 | 1.119 | 36.210 | 0.0369 |
| ΔPain/discomfort | Continuous | 1.746 | 1.181 | 2.581 | 0.0052 | 0.885 | 0.330 | 2.375 | 0.8082 |
| ΔAnxiety/depression | Continuous | 1.485 | 0.817 | 2.698 | 0.1949 | - | - | - | - |
| ΔHealth state | Continuous | 0.983 | 0.963 | 1.003 | 0.1017 | - | - | - | - |
| PFS | Event/ censor | 2.391 | 1.592 | 3.590 | <.0001 | 4.226 | 0.700 | 25.507 | 0.1161 |
| Clinical PFS (until clinical PD) | Event/ censor | 2.595 | 1.357 | 4.962 | 0.0039 | - | - | - | - |

CI: confidence interval; BMI: body mass index; WBC: white blood cell count; ALT: alanine aminotransferase; ALP: alkaline phosphatase; Cr: creatinine; LDH: lactate dehydrogenase; CRP: C-reactive protein; FT4: thyroxine; irAE; immune-related adverse events; PS: performance status; RECIST: Response Evaluation Criteria in Solid Tumors; CR: complete response; PR: partial response; SD: stable disease; PD: progressive disease; NE: not evaluated; PFS: progression-free survival; Δ: changes between baseline to week 25.

**Supplementary Table 7.** Immune-related adverse events from nivolumab initiation to 4 weeks (*n* = 243)

|  | **Grade 1**  ***n* (%)** | **Grade 2**  ***n* (%)** | **Grade 3**  ***n* (%)** | **Grade 4**  ***n* (%)** |
| --- | --- | --- | --- | --- |
| Any | 30 (12.3) | 17 (7.0) | 10 (4.1) | 0 (0.0) |
| Rash acneiform | 7 (2.9) | 4 (1.6) | 1 (0.4) | 0 (0.0) |
| Pruritus | 8 (3.3) | 3 (1.2) | 1 (0.4) | 0 (0.0) |
| Skin hypopigmentation | 0 (0.0) | 0 (0.0) | 0 (0.0) | 0 (0.0) |
| Pneumonitis | 1 (0.4) | 3 (1.2) | 2 (0.8) | 0 (0.0) |
| Pleural effusion | 2 (0.8) | 1 (0.4) | 1 (0.4) | 0 (0.0) |
| Pericardial effusion | 2 (0.8) | 0 (0.0) | 0 (0.0) | 0 (0.0) |
| Hyperthyroidism | 1 (0.4) | 1 (0.4) | 0 (0.0) | 0 (0.0) |
| Hypothyroidism | 1 (0.4) | 2 (0.8) | 0 (0.0) | 0 (0.0) |
| Hypopituitarism | 0 (0.0) | 0 (0.0) | 0 (0.0) | 0 (0.0) |
| Type 1 diabetes | 0 (0.0) | 0 (0.0) | 0 (0.0) | 0 (0.0) |
| Stomatitis | 1 (0.4) | 2 (0.8) | 0 (0.0) | 0 (0.0) |
| Diarrhea | 4 (1.6) | 0 (0.0) | 2 (0.8) | 0 (0.0) |
| Colitis | 1 (0.4) | 1 (0.4) | 1 (0.4) | 0 (0.0) |
| AST elevation | 7 (2.9) | 0 (0.0) | 0 (0.0) | 0 (0.0) |
| ALT elevation | 2 (0.8) | 1 (0.4) | 0 (0.0) | 0 (0.0) |
| gGTP elevation | 4 (1.6) | 0 (0.0) | 0 (0.0) | 0 (0.0) |
| T bilirubin elevation | 1 (0.4) | 0 (0.0) | 0 (0.0) | 0 (0.0) |
| Hepatitis | 2 (0.8) | 0 (0.0) | 0 (0.0) | 0 (0.0) |
| Malaise | 9 (3.7) | 4 (1.6) | 2 (0.8) | 0 (0.0) |
| Edema | 5 (2.1) | 2 (0.8) | 0 (0.0) | 0 (0.0) |
| Anorexia | 9 (3.7) | 1 (0.4) | 2 (0.8) | 0 (0.0) |
| Arthralgia | 2 (0.8) | 1 (0.4) | 0 (0.0) | 0 (0.0) |
| Arthritis | 0 (0.0) | 0 (0.0) | 0 (0.0) | 0 (0.0) |
| Infusion reaction | 1 (0.4) | 2 (0.8) | 0 (0.0) | 0 (0.0) |
| Peripheral sensory neuropathy | 2 (0.8) | 0 (0.0) | 0 (0.0) | 0 (0.0) |
| Central nervous system disorder | 0 (0.0) | 0 (0.0) | 0 (0.0) | 0 (0.0) |
| Myasthenia gravis | 0 (0.0) | 0 (0.0) | 0 (0.0) | 0 (0.0) |
| Others | 8 (3.5) | 2 (0.9) | 2 (0.9) | 0 (0.0) |

AST: aspartate transaminase; ALT: alanine aminotransferase; gGTP: gamma glutamyltransferase.

**Supplementary Table 8.** Immune-related adverse events from weeks 5–8 after nivolumab initiation (*n* = 231)

|  | **Grade 1**  ***n* (%)** | **Grade 2**  ***n* (%)** | **Grade 3**  ***n* (%)** | **Grade 4**  ***n* (%)** |
| --- | --- | --- | --- | --- |
| Any | 18 (7.8) | 16 (6.9) | 3 (1.3) | 2 (0.9) |
| Rash acneiform | 9 (3.9) | 1 (0.4) | 0 (0.0) | 0 (0.0) |
| Pruritus | 3 (1.3) | 1 (0.4) | 0 (0.0) | 0 (0.0) |
| Skin hypopigmentation | 2 (0.9) | 2 (0.9) | 0 (0.0) | 0 (0.0) |
| Pneumonitis | 2 (0.9) | 4 (1.7) | 2 (0.9) | 0 (0.0) |
| Pleural effusion | 1 (0.4) | 0 (0.0) | 0 (0.0) | 0 (0.0) |
| Pericardial effusion | 2 (0.9) | 0 (0.0) | 0 (0.0) | 0 (0.0) |
| Hyperthyroidism | 5 (2.2) | 3 (1.3) | 1 (0.4) | 0 (0.0) |
| Hypothyroidism | 0 (0.0) | 3 (1.3) | 0 (0.0) | 0 (0.0) |
| Hypopituitarism | 0 (0.0) | 1 (0.4) | 0 (0.0) | 1 (0.4) |
| Type 1 diabetes | 0 (0.0) | 2 (0.9) | 0 (0.0) | 0 (0.0) |
| Stomatitis | 1 (0.4) | 0 (0.0) | 0 (0.0) | 0 (0.0) |
| Diarrhea | 2 (0.9) | 0 (0.0) | 1 (0.4) | 0 (0.0) |
| Colitis | 4 (1.7) | 0 (0.0) | 0 (0.0) | 0 (0.0) |
| AST elevation | 5 (2.2) | 1 (0.4) | 0 (0.0) | 1 (0.4) |
| ALT elevation | 6 (2.6) | 2 (0.9) | 0 (0.0) | 0 (0.0) |
| gGTP elevation | 0 (0.0) | 0 (0.0) | 2 (0.9) | 0 (0.0) |
| T bilirubin elevation | 0 (0.0) | 1 (0.4) | 0 (0.0) | 0 (0.0) |
| Hepatitis | 4 (1.7) | 2 (0.9) | 1 (0.4) | 0 (0.0) |
| Malaise | 3 (1.3) | 5 (2.2) | 1 (0.4) | 0 (0.0) |
| Edema | 5 (2.2) | 2 (0.9) | 1 (0.4) | 0 (0.0) |
| Anorexia | 2 (0.9) | 3 (1.3) | 0 (0.0) | 0 (0.0) |
| Arthralgia | 1 (0.4) | 2 (0.9) | 0 (0.0) | 0 (0.0) |
| Arthritis | 0 (0.0) | 0 (0.0) | 0 (0.0) | 0 (0.0) |
| Infusion reaction | 1 (0.4) | 0 (0.0) | 0 (0.0) | 0 (0.0) |
| Peripheral sensory neuropathy | 0 (0.0) | 0 (0.0) | 0 (0.0) | 0 (0.0) |
| Central nervous system disorder | 0 (0.0) | 0 (0.0) | 0 (0.0) | 0 (0.0) |
| Myasthenia gravis | 0 (0.0) | 1 (0.4) | 0 (0.0) | 0 (0.0) |
| Others | 6 (2.6) | 5 (2.2) | 1 (0.4) | 0 (0.0) |

AST: aspartate transaminase; ALT: alanine aminotransferase; gGTP: gamma glutamyltransferase.

**Supplementary Table 9.** Immune-related adverse events overall from initiation of nivolumab (*n* = 243)

|  | **Grade 3**  ***n* (%)** | **Grade 4**  ***n* (%)** |
| --- | --- | --- |
| Any | 21 (8.6) | 1 (0.4) |
| Pruritus | 4 (1.6) | 0 (0.0) |
| Diarrhea | 5 (2.1) | 0 (0.0) |
| Pneumonitis | 11 (4.5) | 1 (0.4) |
| Hypothyroidism | 3 (1.2) | 0 (0.0) |
| Type 1 diabetes | 1 (0.4) | 0 (0.0) |
| Others | 19 (7.8) | 2 (0.8) |
